# An Evaluation of Prognostic Factors in Cutaneous Squamous Cell Carcinoma: A Single-Center Study of 237 Japanese Cases

**DOI:** 10.3390/jcm14041243

**Published:** 2025-02-13

**Authors:** Emi Yamazaki, Taku Fujimura, Manami Takahashi-Watanabe, Ryo Amagai, Erika Tamabuchi, Kenta Oka, Yumi Kambayashi, Akira Hashimoto, Ryoko Omori, Takehiro Takahashi, Yoshihide Asano

**Affiliations:** Department of Dermatology, Tohoku University Graduate School of Medicine, Sendai 980-8575, Japan; hemiura0804@derma.med.tohoku.ac.jp (E.Y.); manami777@derma.med.tohoku.ac.jp (M.T.-W.); amagai@derma.med.tohoku.ac.jp (R.A.); eri-tama@derma.med.tohoku.ac.jp (E.T.); kenta.oka@derma.med.tohoku.ac.jp (K.O.); kambayashi@derma.med.tohoku.ac.jp (Y.K.); hashi-a@med.tohoku.ac.jp (A.H.); shimada@derma.med.tohoku.ac.jp (R.O.); takehiro.takahashi@derma.med.tohoku.ac.jp (T.T.); yasano@derma.med.tohoku.ac.jp (Y.A.)

**Keywords:** cutaneous SCC, hidradenitis suppurativa, prognosis, proinflammatory cytokines, LL-37

## Abstract

**Background/Objectives**: Cutaneous squamous cell carcinoma (cSCC) is a common cutaneous malignancy with diverse etiologies, including actinic keratosis, burns, and hidradenitis suppurativa (HS). **Methods**: We reviewed 237 cases diagnosed as cSCC in our department between 2013 and 2023. In addition, we focused on HS as an aggravating factor for cSCC. The mechanism of cSCC progression was investigated with a focus on LL-37, a peptide implicated in the pathogenesis of HS. Relevant gene expression was analyzed comprehensively via RNA sequencing in vitro. **Results**: The median age of the patients was 82 years (range: 33–101), with 139 males and 98 females. The primary sites were as follows: head and neck (125 cases), trunk (10 cases), vulva (11 cases), extremities (81 cases), and buttocks (10 cases). Among the five cases (2.1%) of buttocks cSSc associated with HS, all cases (100%) had local recurrence or lymph node metastasis at the time of diagnosis, and one case (20%) developed multi-organ metastasis. The incidence of disease progression in cSCC originating from HS-affected buttocks was significantly higher than in other sites (*p* < 0.05). RNA sequencing revealed the significant amplification of *ACTA1*, which was confirmed by Western blotting and immunohistochemical staining. **Conclusions**: These results suggest that HS is a prognostic factor in cSCC and that LL-37 stimulation contributes to tumor progression, partly by enhancing multiple tumor growth factors.

## 1. Introduction

Cutaneous squamous cell carcinoma (cSCC) is a common cutaneous malignancy with diverse etiologies, including actinic keratosis, burns, and hidradenitis suppurativa (HS) [1,2]. cSCC generally has a favorable prognosis in terms of the recurrence rate unless diagnosed at a late stage [3]. In addition, immune checkpoint inhibitor (ICI)-based immunotherapy, such as anti-PD-1 antibodies, has shown promising therapeutic potential for cSCC [4]. This underscores the importance of the tumor microenvironment’s immunological components, although the precise mechanisms remain unknown. On the other hand, cSCC arising from HS exhibits a poor prognosis, but its underlying mechanisms also remain unclear [5].

HS is a neutrophilic autoinflammatory and immune-mediated skin disease, and recent studies have identified cytokines, such as IL-17A/F and members of the IL-1 family, as key factors in its pathogenesis, particularly with the use of biologic agents [6,7]. Alongside these proinflammatory cytokines, cathelicidin (LL-37) has been implicated in HS pathogenesis [8,9]. For example, LL-37 expression is significantly increased in the apocrine sweat glands and the distal outer root sheath of hair follicle epithelium in lesional HS skin compared to healthy controls. Notably, neutrophils abundant in HS lesions are a major source of LL-37, although other cells, such as keratinocytes, also release them [10,11]. Beyond its immunomodulatory effects, LL-37 promotes angiogenesis in the skin by inducing vascular endothelial growth factor (VEGF) and matrix metalloproteinases (MMPs), contributing to the progression of various cancers, including skin cancer [12,13]. Furthermore, LL-37 and MMP expression increase in parallel with the dermal invasion of tumor cells in cSCC [14,15]. In addition, since RNA sequencing (RNA-Seq) can comprehensively analyze gene expression in tumors, it is possible to extensively examine tumor cells’ responses to various external factors [16]. Additionally, RNA sequencing often leads to the discovery of previously unreported factors involved in tumor progression. Based on these findings, we reviewed 237 cases of cSCC from a single center. Furthermore, we investigated the potential mechanisms underlying the poor prognosis of cSCC arising from HS by using RNA-Seq to evaluate the tumor-promoting effects of LL-37 on the A431 human SCC cell line in vivo.

## 2. Materials and Methods

### 2.1. Ethics Statement

The protocol for this study was approved by the ethics committee at Tohoku University Graduate School of Medicine, Sendai, Japan (permit number: 2021-1-1213, approved at 30 March 2022). All procedures were performed in accordance with relevant guidelines and regulations. Written informed consent was obtained from all patients.

### 2.2. Patients

A database maintained by the dermatology departments at Tohoku University Graduate School of Medicine was retrospectively reviewed. A total of 237 patients diagnosed with cSCC between January 2013 and March 2023 were identified (Table 1).

### 2.3. Cell Line and Cell Culture

Human epidermoid squamous carcinoma A431 cell lines were purchased from the American Type Culture Collection (Manassas, VA, USA). Cells were cultured in DMEM (Sigma-Aldrich, St. Louis, MO, USA) supplemented with 10% fetal bovine serum (Biological Industries, Kibbutz Beit Haemek, Israel), penicillin (100 units/mL), streptomycin (0.1 mg/mL), and amphotericin B (0.25 μg/mL). A431 cells were stimulated or unstimulated with 0.3 mM human cathelicidin (LL-37) (Genemed Synthesis Inc., San Antonio, TX, USA) [13]. Cells were harvested 6 h after stimulation for RNA extraction and 48 h after stimulation for Western blot analysis.

### 2.4. RNA Extraction and RNA Sequencing

Since cSCC associated with HS exhibited significantly higher rates of local recurrence or lymph node metastasis than other cSCC cases, we hypothesized that the HS-related immunological background may play a role in the exacerbation of cSCC. LL-37, a molecule known to induce Th17 response, is abundant in HS and has been implicated in its pathogenesis [9]. Based on these findings, we further hypothesized that LL-37 may directly influence SCC cells to promote cancer progression.

To test this hypothesis, we stimulated A431 cells with LL-37 and evaluated its effects using RNA-Seq. Total RNA was extracted using an RNeasy Micro Kit (Qiagen, Courtaboeuf, France) according to the manufacturer’s instructions. RNA was eluted with 14 μL of RNase-free water, and DNase I treatment (RNase-Free DNase Set; Qiagen) was performed to eliminate genomic DNA contamination. Reverse transcription was conducted using the SuperScript VILO cDNA Synthesis Kit (Invitrogen, Carlsbad, CA, USA). RNA sequencing (RNA-Seq) was outsourced to AZENTA Ltd., Tokyo, Japan.

### 2.5. Western Blotting

A431 cells were seeded in 6-well plates and cultured as described above. Cells were harvested and lysed in lysis buffer (Cell Signaling Technology, Boston, MA, USA). After the addition of SDS sample buffer (Cell Signaling Technology), the lysates were subjected to electrophoresis on 12% polyacrylamide gel (ATTO Corp., Tokyo, Japan). Proteins were transferred to a polyvinylidene difluoride membrane (Bio-Rad, Hercules, CA, USA). The membrane was blocked with 5% nonfat dry milk in Tris-buffered saline with 0.1% Tween-20 (TBST) for 1 h at room temperature. Following several washes with TBST, the membrane was incubated overnight at 4 °C with a mouse anti-human ACTA1 primary antibody (R&D Systems, Minneapolis, MN, USA; 1:1000) or a mouse anti-human beta-actin antibody (Cell Signaling Technology, Tokyo, Japan; 1:1000). After additional washes with TBST, the membrane was incubated with horseradish peroxidase-conjugated goat anti-mouse IgG secondary antibody (Santa Cruz Biotechnology, Dallas, TX, USA) for 1 h.

### 2.6. Tissue Samples and Immunohistochemical Staining

Immunostaining was performed on three randomly selected cases of cSCC derived from HS from five cases and on three cases of cSCC of the trunk or buttocks (with the same site onset of cSCC derived from HS) without inflammatory disease from fifteen cases. Formalin-fixed, paraffin-embedded skin specimens from the initial visits of three patients with cSCC, with or without HS, treated at the Department of Dermatology, Tohoku University Graduate School of Medicine, were analyzed. Rabbit polyclonal antibodies for human ACTA1 (Proteintech, Tokyo, Japan) were used for immunohistochemical (IHC) staining.

### 2.7. Statistical Analysis

Fisher’s exact test was used to compare recurrence rates between groups. All statistical analyses were performed using GraphPad Prism 9.5.1.733. A *p*-value of <0.05 was considered statistically significant.

## 3. Results

### 3.1. Demographic Data

**Figure 1:**
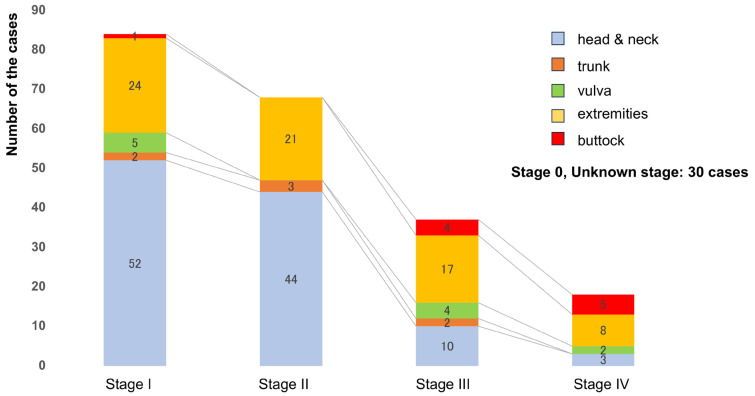
**Clinical sites of cSCC at each stage.** The sites of presentation were as follows: head and neck in 125 cases, trunk in 10 cases, vulva in 11 cases, extremities in 81 cases, and buttocks in 10 cases, respectively.

The demographic characteristics of the patients are summarized in Table 1. The cohort included 139 men and 98 women, with a median age of 82 years. The anatomical distribution of cSCC was as follows: head and neck (125 cases, 52.7%), trunk (9 cases, 3.8%), extremities (81 cases, 34.2%), external genitalia (11 cases, 4.6%), and buttocks (11 cases, 4.6%). Among the 237 patients, 183 underwent radical excision, while 54 were treated at other institutions prior to presentation at our center (Figure 1). The clinical stages of cSCC were distributed as follows: 7 patients (3%) had stage 0, 83 (35%) had stage I, 68 (29%) had stage II, 37 (16%) had stage III, 18 (8%) had stage IV, and 23 (10%) had an unknown stage. The proportion of cSCC cases located on the buttocks was 1.2% in stage I, 0% in stage II, 11% in stage III, and 28% in stage IV.

The demographic characteristics of the cSCC derived from HS are summarized in Table 2. The median Sartorius score is 53 (42–97). Three cases developed local recurrence, and two cases developed lymph node metastasis.

### 3.2. Recurrence Rates by Site

Of the 125 head and neck cSCC cases, 11 (8.8%) experienced local recurrence or lymph node metastasis. No recurrence or metastasis was observed in the 9 trunk cases, while 1 out of 11 vulvar cases (9.1%) and 5 out of 81 extremity cases (6.2%) showed recurrence or metastasis. In contrast, local recurrence or lymph node metastasis occurred in 9 out of 11 buttocks cSCC cases (81.8%). Notably, the co-occurrence of HS was 55% among buttocks cSCC cases, and all of these cases (100%) exhibited local recurrence or lymph node metastasis (Table 1 and Table 2). Fisher’s exact test revealed a significantly higher rate of local recurrence or lymph node metastasis in buttocks cSCC compared to cSCC at other anatomical sites (*p* < 0.05). Furthermore, buttocks cSCC associated with HS demonstrated significantly higher recurrence or metastasis rates than other types of cSCC (*p* < 0.05).

### 3.3. A Comprehensive Analysis of the Cancer-Promoting Effects of LL-37 on mRNA Expression and Protein Production in A431 Human SCC Cells

**Figure 2:**
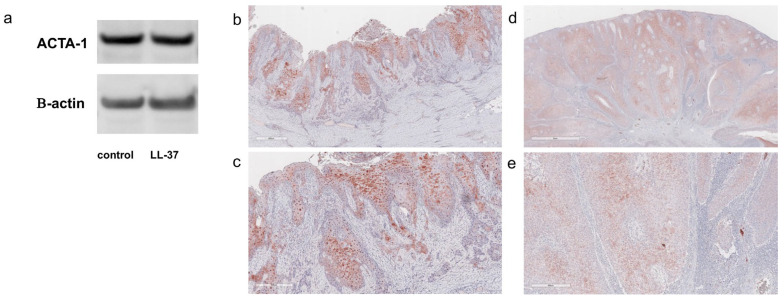
**ACTA1 production in cSCC.** ACTA1 production was detected by Western blotting (**a**). Immunohistochemical staining of ACTA1 in cSCC (**b**–**e**). Formalin-fixed, paraffin-embedded specimens were stained with anti-rabbit ACTA1 antibodies: cSCC developing from HS (**b**,**c**) and cSCC without HS (**d**,**e**).

We performed a GO analysis to annotate the functions of host genes associated with the differentially expressed genes, identifying the top 18 enriched GO terms. These included terms related to biological processes, such as muscle system processes, collagen fibril organization, the regulation of cell–substrate adhesion, epithelial cell differentiation, heterophilic cell–cell adhesion via plasma membrane cell adhesion molecules, response to estrogen, response to wounding, response to extracellular stimuli, and the regulation of body fluid levels. A comprehensive RNA-Seq analysis revealed significant mRNA upregulation of several genes, including *ACTA1*, *IGFBP2*, *MUC5B*, *CEACAM5*, and *KRT19*, all of which are associated with cancer progression in various types of malignancies (Table 3; *p* < 10^−10^). To validate the increased mRNA expression of *ACTA1* in A431 cells, the protein production of ACTA1 was examined by Western blotting, which confirmed its upregulation (Figure 2a).

### 3.4. Immunohistochemical Staining of ACTA1 in cSCC

To confirm the increased expression of ACTA1 in cSCC, we performed IHC staining on cSCC tissues associated with HS and those without HS. ACTA1 was strongly expressed in the cytoplasm of cSCC cases associated with HS (Figure 2b,c), whereas its expression in cSCC without HS was patchy and punctate (Figure 2d,e).

## 4. Discussion

HS is a neutrophilic autoinflammatory and immune-mediated skin disease that can serve as an origin for cSCC. Although cSCC arising from HS is known to have a poor prognosis, the mechanisms underlying its carcinogenesis remain unclear [17]. TNF-α inhibitors, such as adalimumab, used in the treatment of HS, are not associated with an increased risk of developing cSCC, suggesting that TNF-α is not directly involved in cSCC development [18]. On the other hand, the efficacy of IL-17A/IL-17F inhibitors [6] and IL-1 inhibitors [7] in the treatment of HS has recently been demonstrated in several clinical trials. In addition, previous studies have shown that an RNA-Seq analysis of HS revealed increased levels of Th17-related cytokine signaling in patients with HS [19]. These findings indicate the potential involvement of IL-17A/IL-17F and IL-1 in the pathogenesis of HS.

In the context of cSCC carcinogenesis, IL-17 signaling in keratinocytes has been shown to promote sustained activation of the IL-17-dependent RAF4-ERK5 axis, leading to keratinocyte proliferation and tumor formation [20]. Furthermore, aryl hydrocarbon receptor (AhR)-mediated IL-17/IL-1 family signaling has been implicated in the progression of cSCC in murine models [21]. LL-37, a known activator of NLRP3 inflammasomes, has been shown to induce IL-17- and IL-1β-mediated inflammation in HS, and the inhibition of NLRP3 inflammasomes can reduce this inflammatory response [22]. Neutrophils are a major source of LL-37 [10], and LL-37 is abundant in HS lesions [9]. Notably, the serum levels of LL-37 have been reported to correlate with IL-17 levels in patients with HS [8]. Moreover, several studies have demonstrated that LL-37 is associated with poor prognosis in various cancers, including cSCC [23,24,25]. Together, these findings suggest that the LL-37/IL-1/Th17 axis may play a pivotal role in the pathogenesis of cSCC arising from HS.

In our cohort, 55% of patients with cSCC located on the buttocks were found to be associated with HS. In fact, Fisher’s exact test showed a statistically significant higher incidence of cSCC in tumors located on the buttocks to be concomitant with the HS group. The recurrence rate of cSCC located on the buttocks with HS was significantly higher (100%), while the recurrence rates of cSCC located on the head and neck, trunk, vulva, and extremities were 8.8%, 0%, 9.1%, and 6.2%, respectively, suggesting that the complication of HS is closely associated with the development and progression of cSCC. Moreover, an in vitro analysis revealed that the A431 human SCC cell line stimulated by LL-37 increased the mRNA expression of ACTA1, which is reported to regulate the initiation and progression of head and neck SCC [23]. Notably, the expression of ACTA1 in tumor cells is higher in cSCC arising from HS compared to cSCC on the trunk without HS. Interestingly, ACTA1 is one of the biomarkers used to predict the prognosis of head and neck SCC, along with plasminogen activator inhibitor-1 and urokinase-type plasminogen activator [24]. In another report, ACTA1 was elevated in oral SCC with neural invasion and a lower ratio of CD3+CD8+ cytotoxic T cells, resulting in a poor prognosis among subsets of oral SCC [26]. Although there are no English reports on the role of ACTA1 expression in cSCC, increased ACTA1 expression may indicate a poor prognosis in cSCC developing from HS. HS-derived LL-37 stimulates tumor cells and increases the expression of ACTA1, which may be related to the poor prognosis of cSCC arising from HS.

This study has several limitations. The relatively small sample size, especially in patients with cSCC associated with HS, limits the generalizability of the findings. Larger cohorts are needed for confirmation. Additionally, the observational nature of this study prevents us from establishing causality between the LL-37/IL-1/Th17 axis and cSCC progression, and the specific roles of IL-1 and Th17 cytokines require further exploration. Experimental and prospective studies are needed to clarify their contributions to cSCC development, particularly in the context of HS.

## 5. Conclusions

In summary, our study highlights the significant role of HS as a prognostic factor in cSCC development, demonstrating that cSCC arising from HS, especially on the buttocks, is associated with a higher risk of local recurrence, lymph node metastasis, and poor prognosis. We also reveal the potential of LL-37 in promoting tumor progression via the upregulation of cancer-related genes, such as ACTA1. These findings underscore the importance of considering HS in the management of cSCC, with LL-37 emerging as a potential therapeutic target to mitigate the aggressive nature of cSCC in these patients.

The limitation of this study is that, although the results of the RNA-Seq analysis provide valuable insights, a clear correlation with clinical outcomes has not been demonstrated. Further in vivo research is needed to investigate the therapeutic potential of targeting the LL-37/IL-1/Th17 axis in preventing cSCC progression in patients with inflammatory skin conditions like HS.

## Figures and Tables

**Table 1:** Demographic characteristics of patients with cSCC.

Age (Mean)	
Whole	82 (33–101)
Male	79 (43–98)
Female	82 (33–101)
Sex	
Male	139 (58.6%)
Female	98 (41.4%)
Location	
Head and neck	125 (52.7%)
Trunk	9 (3.8%)
External genitalia	11 (4.6%)
Extremities	81 (34.2%)
Buttocks	11 (4.6%)
Recurrence/lymph node metastasis	
Head and neck	11 (8.8%)
Trunk	0
External genitalia	1 (9.1%)
Extremities	5 (6.2%)
Buttocks	9 (81.8%)
Hidradenitis supprativa	5 (100%)
Clinical stage (UICC ver.8)	
0	7 (3.0%)
I	84 (35.4%)
II	68 (28.7%)
III	37 (15.6%)
IV	18 (7.6%)
Unknown	23 (9.7%)

**Table 2:** Primary tumor size, stage, recurrence style, and HS score.

	Age	Sex	Primary Tumor (mm)	Stage	Recurrence	Duration of HS	Sartorius Score
Case 1	70	male	30 × 30	T2N0Mo stage II	local	20 years	10
Case 2	55	male	140 × 100	T4aN0Mo stage IVA	local	45 years	56
Case 3	72	male	120 × 100	T3N0M0 stage III	lymph node	50 years	57
Case 4	72	male	N.A.	T4aN0Mo stage IVA	lymph node	40 years	64
Case 5	48	male	170 × 150	T3N0M0 stage III	local	20 years	97

N.A.: not assessed.

**Table 3:** List of top 10 differentially expressed genes (DEG) in A431 stimulated with LL-37.

Gene ID	Gene Name	Start	End	Strand	logFC	logCPM	*p* Value	FDR	Expression
ENSG00000143632	ACTA1	229,000,000	2.29 × 10^8^	−	5.710901	0.703475	1.08 × 10^−26^	3.54 × 10^−22^	UP
ENSG00000125414	MYH2	10,521,148	10,549,700	−	8.314106	0.192412	1.69 × 10^−22^	2.21 × 10^−18^	UP
ENSG00000115457	IGFBP2	217,000,000	2.17 × 10^8^	+	8.760441	2.921457	1.99 × 10^−20^	1.86 × 10^−16^	UP
ENSG00000117983	MUC5B	1,223,066	1,262,172	+	10.56984	2.440878	1.67 × 10^−19^	1.21 × 10^−15^	UP
ENSG00000105388	CEACAM5	41,708,585	41,730,433	+	5.994973	2.886276	7.27 × 10^−16^	4.76 × 10^−12^	UP
ENSG00000171345	KRT19	41,523,617	41,528,308	−	5.412363	3.77336	1.34 × 10^−15^	7.98 × 10^−12^	UP
ENSG00000022267	FHL1	136,000,000	1.36 × 10^8^	+	2.68773	0.724909	2.31 × 10^−15^	1.26 × 10^−11^	UP
ENSG00000047457	CP	149,000,000	1.49 × 10^8^	−	6.025995	1.612597	4.48 × 10^−13^	2.25 × 10^−9^	UP
ENSG00000268104	SLC6A14	116,000,000	1.16 × 10^8^	+	8.679165	0.622586	8.66 × 10^−12^	4.05 × 10^−8^	UP
ENSG00000108515	ENO3	4,948,092	4,957,131	+	1.538244	1.691046	1.01 × 10^−11^	4.42 × 10^−8^	UP

## Data Availability

The data that support the findings of this study are available upon request from the corresponding author, T.F. The data are not publicly available due to them containing information that could compromise the privacy of the research participants.
